# X-Ray Work in a Base Hospital in France: A Few Observations of a Practical Nature

**Published:** 1918

**Authors:** 


***X-Ray Work in a Base Hospital in France : a Few Observations of a Practical Nature.*** By B. H. Stanley Aylward, Captain, R. A. M. C. Abs. from the *Jour. R. A. M. C.*, May 1918.

In war work, the author considers rapidity in handling cases of prime importance. The worker must combine speed and accuracy, and deliver the patient to the surgeon with information given in the most practical manner possible.

The table should be arranged so that the patient need not be removed from the stretcher. A tube box should be placed under the table in such a manner as to be easily moved, preferably by the feet.

Some form of apparatus for taking lateral head plates is advisable. The author recommends such an apparatus devised by Major Higham Cooper.

When possible the foreign body should be clearly indicated by marks on the skin. Plates may be taken in addition if desired but the marks are more serviceable to the surgeon in ordinary cases. Three colors should be used in making the marks : green for antero-posterior, red for lateral, and blue for the nearest point to the skin.

The tube should be carefully centered so that the ray passes upwards through the exact center of the diaphragm.

It is usually best to make the lateral mark first, for the second antero-posterior mark often involves moving the patient. The blunt end of the marker is placed against the skin laterally opposite the foreign body at about the supposed depth. The tube is now moved longitudinally and the movement of the foreign body and the end of the marker compared. Finally a position is found where the end of the marker and the foreign body move together when the tube is moved. The ray is then produced and the point marked with red. An antero-posterior mark is then made in precisely the same way, but with the marker underneath the patient. It is usually necessary to turn the patient or to lift his limb in order to make this mark.

The practice acquired in judging whether the point of the marker is above or below the depth of the foreign body is very useful in the removal of bodies under the rays.

Foreign bodies lying deep in the trunk are best marked either on the back or front in the antero-posterior position, and the depth then worked out by stereoscopic plates.

In head cases antero-posterior and lateral plates are usually sufficient, but it is often difficult to determine from them whether the foreign body is in the skull or not.

For eye localization the author recommends an apparatus evolved from Major Cooper's head apparatus. This is used in connection with circular marker.

